# The Goodness-of-fit of the Fractal Dimension as a Diagnostic Factor in Breast Cancer

**DOI:** 10.7759/cureus.3630

**Published:** 2018-11-24

**Authors:** Sotirios Maipas, Afroditi Nonni, Ekaterini Politi, Helen Sarlanis, Nikolaos G Kavantzas

**Affiliations:** 1 Pathology, School of Medicine, National and Kapodistrian University of Athens, Athens, GRC

**Keywords:** fractal dimension, box-counting dimension, goodness-of-fit, breast cancer, cancer diagnosis

## Abstract

A large number of studies have found that the fractal dimension increases with the progression towards pathological or more pathological states, but there are also studies that have demonstrated the opposite relationship. In this study, we calculate the nuclear box-counting fractal dimension of 109 malignant, 113 benign, and 80 normal isolated breast cells in order to investigate its possible diagnostic importance. We computed the fractal dimension and its goodness-of-fit (i.e., the r-squared value that describes how well the regression line fits the set of the measurements) for two different sets of box size lengths. The statistical analysis did not confirm an important diagnostic potential of the nuclear fractal dimension of isolated breast cells. However, the goodness-of-fit did display a diagnostic potential. The r-squared value may be able to serve as a complementary diagnostic parameter.

## Introduction

Fractal geometry, introduced by the Polish-born French-American mathematician, Benoît B. Mandelbrot, in the 70's, provides us with the necessary geometrical tools to describe the irregular shapes found in nature [1-2]. Fractal dimension is a term of fractal geometry that can be defined as a unitless measure of morphological complexity [3-5]. The box-counting dimension is the most popular and easiest to calculate the fractal dimension, and it can be computed for both fractal and non-fractal objects [3-4, 6-7]. Fractal analysis has been applied in the study of various malignant tumors, such as breast cancer, endometrial carcinoma, and oral and laryngeal cancer [4, 8-14]. A large number of studies have found that the fractal dimension increases with the increase of malignancy, but there are also studies that have demonstrated the opposite relationship [4, 10, 15-16]. Herein, we calculate the nuclear box-counting fractal dimension of isolated malignant, benign, and normal breast cells in order to investigate its possible diagnostic importance.

## Materials and methods

**Figure 1:**
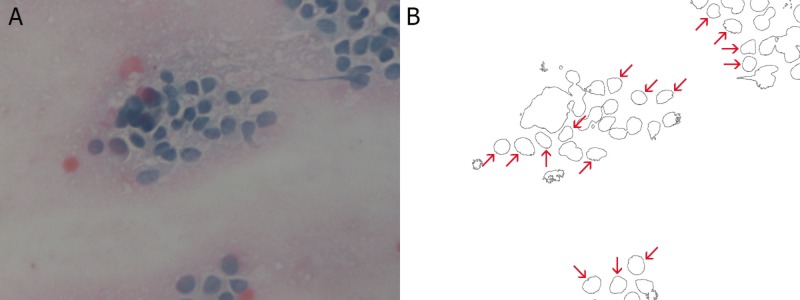
Breast smear of malignant cells from a case of breast adenocarcinoma at 40x magnification (A) and the same image after the necessary transformations (B)

**Figure 2:**
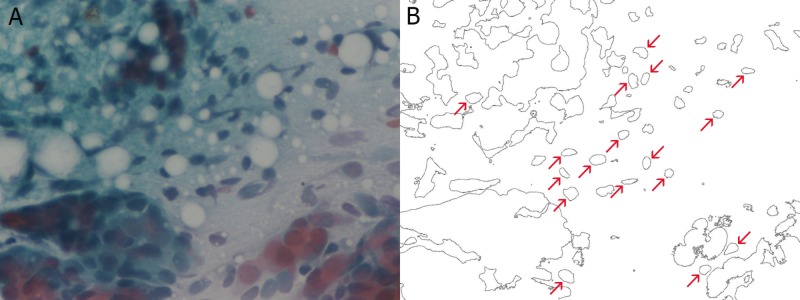
Breast smear of benign cells from a case of fibroepithelial tumor at 40x magnification (A) and the same image after the necessary transformations (B)

**Figure 3:**
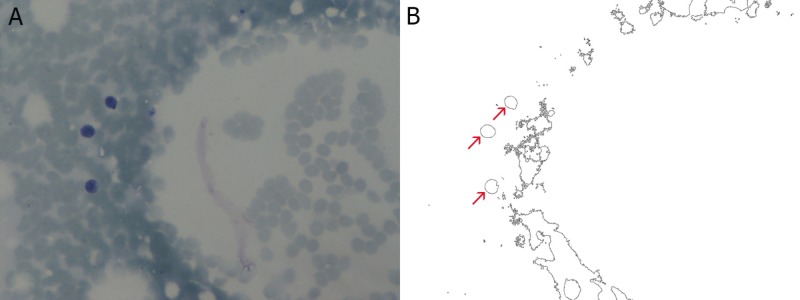
Breast smear of normal epithelial cells at 40x magnification (A) and the same image after the necessary transformations (B)

Three hundred and two cells were selected from 155 electron microscopy images (40x) of breast smears. One hundred and nine cells were malignant, 113 cells were benign, and 80 cells were normal. Each image was introduced into Mathematica 10.4 (Wolfram Research, Champaign, IL) in order to be transformed by built-in Mathematica functions into binary-outline figures, as can be seen in Figures 1-3, where the red arrows indicate the selected nuclei. 

The nuclear box-counting fractal dimension of the selected nuclei and its goodness-of-fit were computed using the open-source plug-in, FracLac, of the ImageJ software (United States National Institute of Health). FracLac covered each nucleus with consecutive square boxes of various side lengths and counted the smallest number of boxes of each size required to cover each nuclear contour. The box-counting fractal dimension was equal to the slope of the regression line of the log-log plot of the scale (scale = box size/image size) and of the number of the boxes [3, 17]. The box size lengths were chosen to be 3, 5, 7, 9, 11, 13, 15, 17, and 19 pixels (Case A), and also 1 to 20 pixels (Case B). The goodness-of-fit of the regression line (i.e., the r-squared value that describes how well the regression line fits the set of the measurements) was also computed by FracLac. All the obtained data were analyzed using the Statistical Package for Social Sciences (SPSS) Statistics, version 20 (IBM SPSS Statistics, Armonk, NY). The statistical analysis included the one-way analysis of variance (ANOVA) and post hoc tests.

The protocol of the study was approved by the Bioethics Committee of the National and Kapodistrian University of Athens, Greece. Furthermore, images were already archived into folders which did not include personal information. Given the fact that we analyzed cells from unknown human subjects, there was no ethical conflict.

## Results

For Case A, the mean fractal dimensions of malignant, benign, and normal cells were 1.123648 ± 0.0589598, 1.146548 ± 0.0706589, and 1.110653 ± 0.0543317, respectively. Statistical analysis revealed a significant difference in the mean fractal dimension of benign and normal cells.

For Case B, the mean fractal dimensions of malignant, benign, and normal cells were 1.072341 ± 0.0400440, 1.086766 ± 0.0448004, and 1.072745 ± 0.0881955, respectively. Contrary to the previous case, the current statistical analysis did not show any significant difference between the three cell groups.

Regarding the goodness-of-fit (r-squared value) for Case A, the mean values were 0.991072 ± 0.0068385 for malignant cells, 0.983985 ± 0.0134711 for benign cells, and 0.986498 ± 0.110298 for normal cells. The post hoc tests revealed a significant difference in the mean values between malignant and benign cells.

For Case B, the mean r-squared values were 0.992738 ± 0.0042910 for malignant cells, 0.987791 ± 0.0070012 for benign cells, and 0.989768 ± 0.0065955 for normal cells. Post hoc tests revealed significant differences in the mean values between malignant and benign cells, as well as between malignant and normal cells.

In both cases, the statistical analysis revealed that the goodness-of-fit of the fractal dimension may display diagnostic potential, as it was able to distinguish malignant from benign cells.

## Discussion

It has been demonstrated that fractal properties are altered with development, growth, aging, and also in disease [18]. However, it is difficult to establish a general rule about the behavior of the fractal dimension in regard to progression towards pathological (or more pathological) states.

Our study demonstrated that the goodness-of-fit of the fractal dimension, known for its prognostic potential [19-21], may serve as a complementary diagnostic parameter. We also confirmed the fact that the box-counting method for the calculation of the fractal dimension is box-size sensitive [22].

An important limitation for the calculation of nuclear box-counting fractal dimensions of isolated cells arises from the overlapping nuclei in smears which decrease the level of automatization of the procedure or make many images inappropriate for the calculation of nuclear fractal dimensions. Moreover, if the experimental dataset includes lobular breast cancer cells (as in our study), the study of morphological characteristics requires more attention because lobular malignant cells may have small nuclei and, therefore, sometimes may not be easily distinguishable from non-malignant cells [23].

Moreover, the digital camera specifications, the quality (e.g., resolution, noise) of the acquired images, the quality of the segmentation (procedure, aiming at separating the regions of interest from the rest of the image, which depends on the thresholding method), and the software tools affect the fractal dimension values [24-26]. The existence of noise impedes the full automatization of the process because, for instance, final (binary) images may have unnecessary objects that must be removed by the user before the calculation of the fractal dimension. It should also be added that the method of outlining in the relevant literature is not always automatic, as outlining has been performed with the help of pointer or by hand [11, 14, 27].

There is no doubt that more extensive research needs to be undertaken regarding the biomedical application of the toolbox of fractal geometry, aiming at increasing its efficacy and its reliability. Given the continuous technological advances in imaging technologies, such as better cameras and software, we expect that fractal analysis is going to be applied more widely.

## Conclusions

Our study used a large number of breast cells. We did not confirm an important diagnostic potential of the nuclear fractal dimension of isolated breast cells. However, the goodness-of-fit did display a diagnostic importance. The r-squared value may be able to serve as a complementary diagnostic parameter.
